# Isolated anterior mitral valve leaflet cleft repair with minimally invasive cardiac surgery using ORBEYE^TM^

**DOI:** 10.1186/s44215-023-00034-5

**Published:** 2023-06-05

**Authors:** Masato Hayama, Mizuki Sumi, Mau Amako, Kiyoyuki Eishi, Hideichi Wada

**Affiliations:** 1 Department of Cardiovascular Surgery, Hakujyuji Hospital, Ishimaru 4-3-1, nishi-ku, Fukuoka-shi, Fukuoka, 819-8511 Japan; 2 Department of Cardiovascular Surgery, Fukuoka University Faculty of Medicine, Fukuoka, Japan

**Keywords:** Isolated anterior mitral leaflet cleft, Minimally invasive cardiac surgery, Mitral valve plasty, ORBEYE^TM^

## Abstract

The cleft mitral valve leaflet is a common cause of congenital mitral regurgitation and is most often associated with other congenital heart defects, usually endocardial cushion defects. Cases in which cleft mitral valve leaflet is found without an associated defect are called isolated mitral valve clefts. We report a case of mitral valve repair for severe MR due to an isolated cleft with minimally invasive cardiac surgery using ORBEYE^TM^ in a 16-year-old male patient. Mitral regurgitation caused due to a defect in the anterior leaflet of the isolated mitral valve was observed on echocardiography. Minimally invasive cardiac surgery was performed using ORBEYE^TM^, a newly developed surgical microscope, and the cleft was closed using interrupted sutures. The postoperative course was uneventful, with minimal mitral regurgitation observed. Therefore, early intervention should be considered for severe mitral regurgitation due to a mitral valve leaflet cleft.

## Background

The cleft mitral valve leaflet (CMVL) is a common cause of congenital mitral regurgitation (MR). CMVL results from congenital mitral hypoplasia and is often associated with endocardial cushion defects [1–4]. CMVL without an associated defect is called an isolated mitral valve cleft, a rare congenital anomaly that was first described in 1954 [5]. Here we report a case of an isolated mitral valve leaflet repair for severe MR due to isolated cleft by minimally invasive cardiac surgery (MICS) using ORBEYE^TM^.

## Case presentation

The patient was a 16-year-old male. A heart murmur was detected during childhood. Echocardiography showed MR, and the patient was subsequently monitored. Severe MR was observed along with expanded left ventricle and atrium. The patient was referred to our department for surgical treatment.

**Fig. 1:**
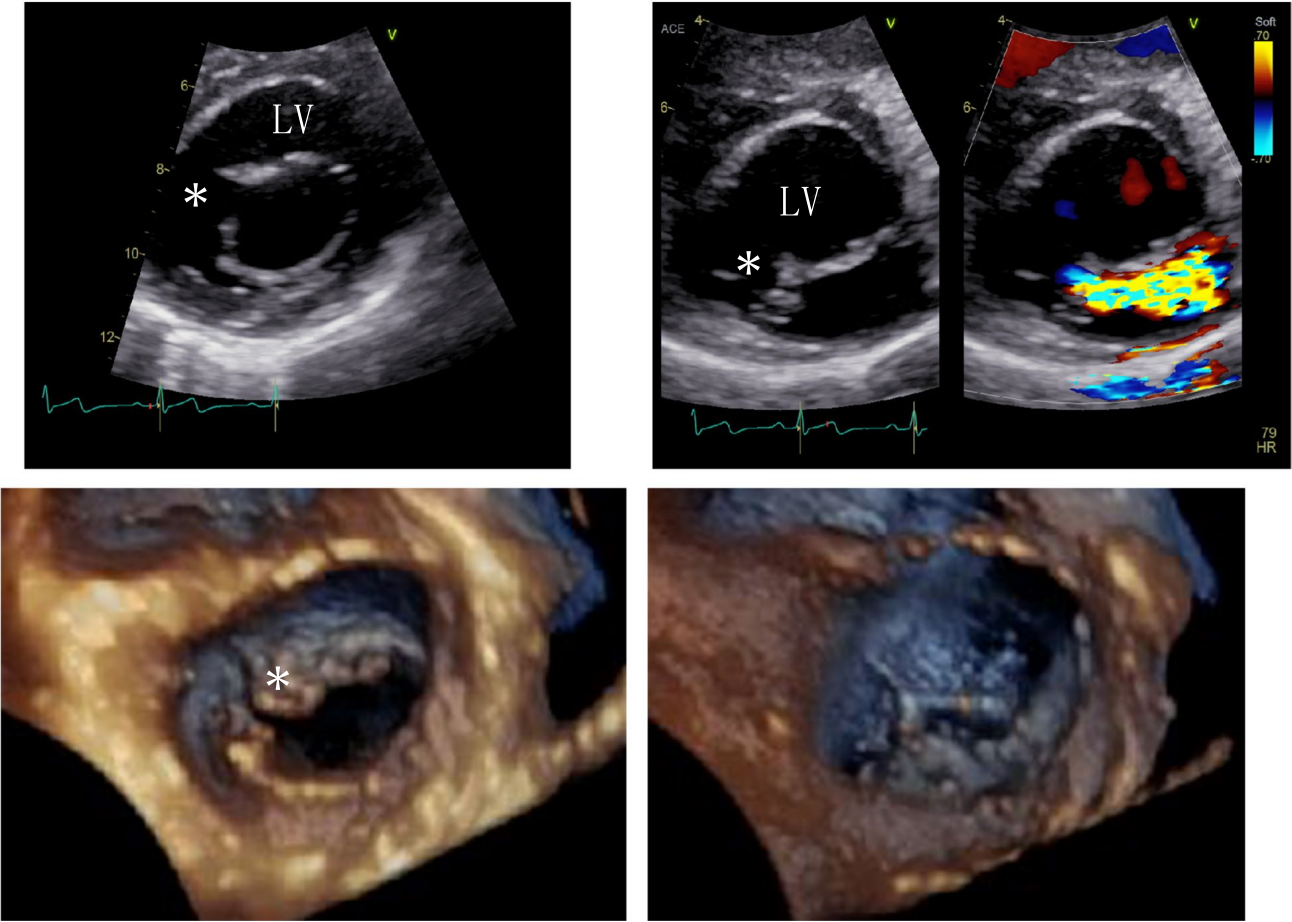
Two-dimensional transthoracic echocardiography (parasternal short-axis view) in a patient with isolated anterior cleft of the mitral valve (*). A mitral regurgitation jet is obtained from the isolated anterior cleft of the mitral valve leaflet

Echocardiography revealed a defect in the anterior leaflet of the isolated mitral valve causing MR (Fig. 1). The mitral annulus was in a normal position, with the cleft pointed toward the left ventricular outflow tract. The mitral and tricuspid valves were attached to the septum at different levels, with the tricuspid valve attached more inferiorly.

We performed MICS using ORBEYE^TM^ (OLYMPUS, Tokyo, Japan). First, we inserted a venous drainage cannula from the right femoral and internal jugular vein and an arterial cannula from the right femoral artery. An incision was made in the right chest 6cm to access the intercostal space. We deflated the right lung and opened the pericardium horizontally superior to the phrenic nerve. We inserted an antegrade cardioplegia catheter into the ascending aorta and performed aortic cross-clamping.

**Fig. 2:**
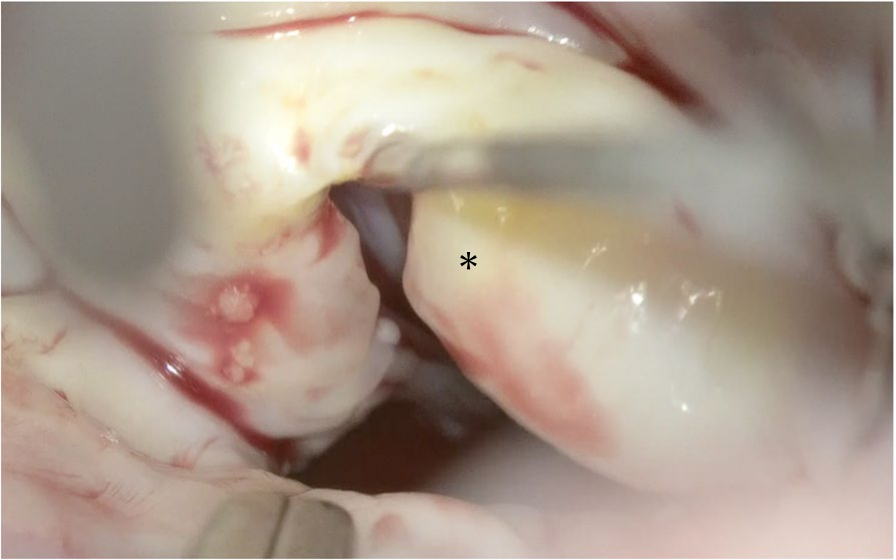
Intraoperative view of an isolated cleft of the anterior leaflet of the mitral valve

**Fig. 3:**
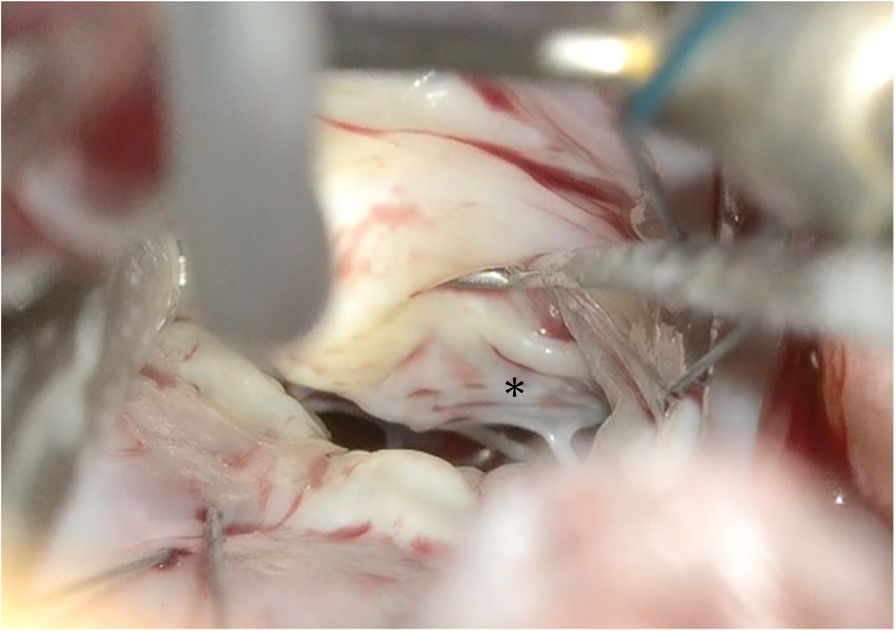
The chordae tendineae at A2 are missing. The chordae tendineae at A3 are membranous

**Fig. 4:**
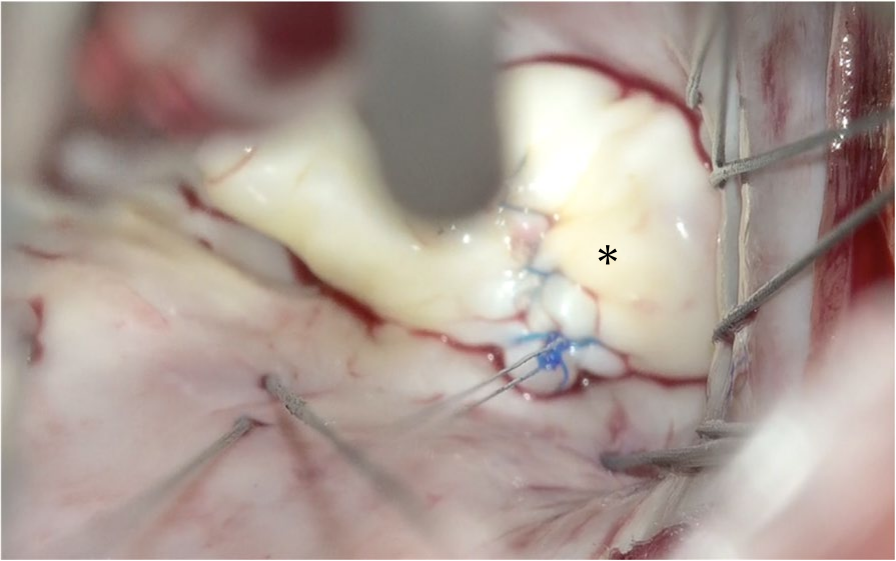
Cleft closure was performed using interrupted sutures with 5-0 Prolene

**Fig. 5:**
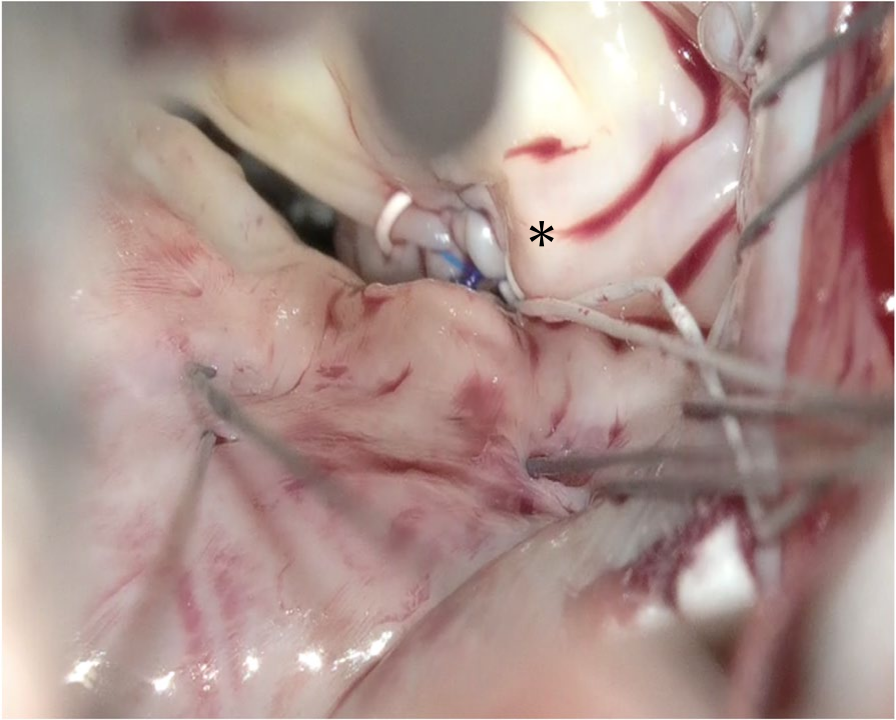
An artificial chordae tendineae was erected from A2 to the posterior papillary muscle

**Fig. 6:**
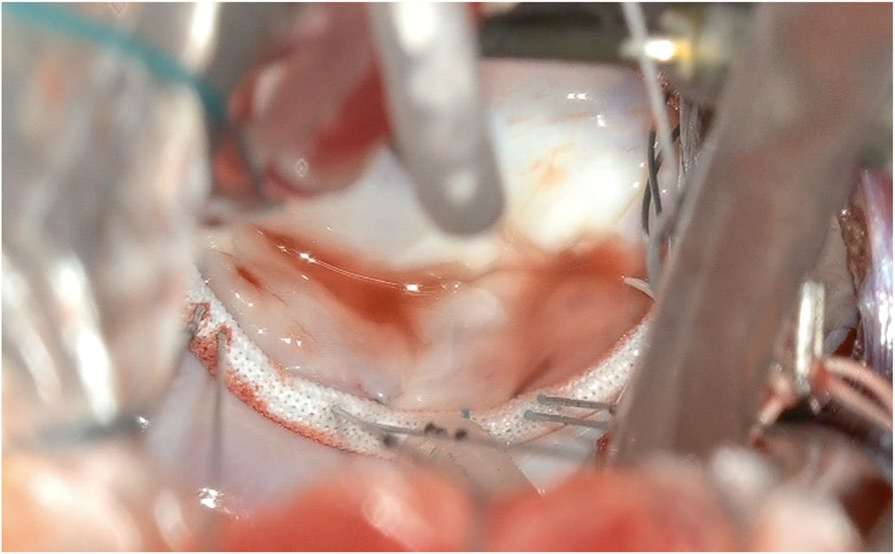
An annuloplasty was performed using an artificial ring and a water test was performed to confirm the absence of leakage

The left atrium was opened and an atrial lift retractor was used to expose the mitral valve. We observed an I-shaped cleft with thin and smooth edges between A2 and A3 segments (Fig. 2). The chordae tendineae were missing at cleft and those in the A3 region were membranous (Fig. 3). The cleft was closed using interrupted sutures with 5-0 Prolene (Fig. 4). Artificial chordae tendineae were implanted from the same site to the posterior papillary muscle (Fig. 5). Annuloplasty was performed using an artificial ring CG future 30mm(Medtronic, Dublin, Ireland) and a water test was performed to check for leakage (Fig. 6). Finally, the cardiopulmonary bypass was withdrawn to complete the surgery. The duration of the operation was 275 min, cardiopulmonary bypass duration was 131 min, and aortic cross-clamping duration was 91 min.

The postoperative course was uneventful, and the echocardiography showed minimal MR. He was discharged from the hospital 16 days after the operation.

## Discussion

**Fig. 7:**
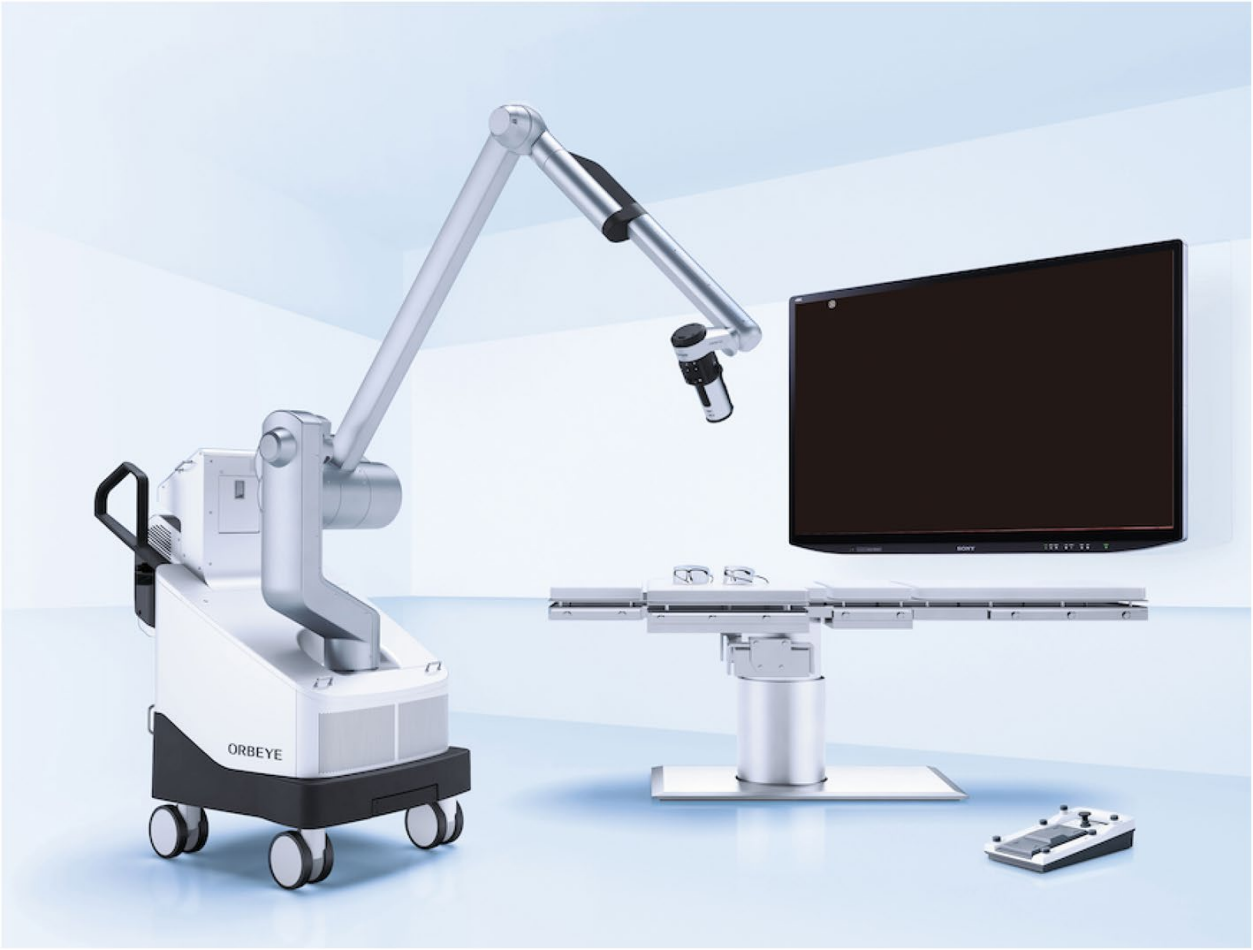
ORBEYE system offers 4K, high-quality, three-dimensional imaging for surgeons. Since it lacks an eyepiece, the surgeons operate while watching surgical field images on a 55-inch monitor using special three-dimensional glasses

A newly developed surgical microscope, ORBEYE^TM^ was launched in the United States and Japan in October 2017. This microscope offers 4K, high-quality, three-dimensional imaging for surgeons. Since it lacks an eyepiece, the surgeons operate while watching surgical field images on a 55-inch monitor using special three-dimensional glasses (Fig. 7) [6]. ORBEYE enables surgery with high image quality and a large screen, making it possible to share intraoperative findings not only with the operator but also with everyone in the operating room. In addition, as an advantage in minimally invasive cardiac surgery, the angle of the camera can be adjusted at hand, so the operative field can be observed at an angle that cannot be seen under direct vision.

The mitral valve leaflet cleft is often associated with congenital heart diseases, such as atrioventricular sepsis. Isolated mitral valve leaflet cleft, such as the one in this case, is rare [1–4]. The mitral valve is known to originate from endocardial cushioning. The atrioventricular tube located between the left side of the primitive atrium and the left ventricular primordium gradually moves to the right. At the same time, the endocardial cushion tissue develops vertically and horizontally, and the atrioventricular tube deforms into an H-shape. As the movement to the right progresses, new traffic is created between the right primitive atrium and ventricle. Eventually, the upper and lower endocardial cushion tissues are fused to form a tricuspid and mitral valve from one atrioventricular canal [7]. The cause of the isolated mitral valve leaflet cleft, as in this case, has not been clarified, but it is expected to be caused by a defect in the endocardial cushion [7].

In this case, since the width of the cleft was small and its edges relatively thin and smooth, it was closed using interrupted sutures. However, if the cleft is relatively large, the cleft is shape like an inverted V, and We are required to repair using glutaraldehyde-treated autologous pericardium for patch material. Di Segni and Edwards reported that mitral valve leaflet clefts in young patients were able to close using interrupted sutures [8]. They observed that a relationship exists between the age of the patients and the thickness of the cleft edges. And there have been many successful outcomes of mitral valve leaflet cleft repair [9]. Hence, early intervention should be considered in case of severe MR due to a mitral valve leaflet cleft. However, in young patients, all organs, including the heart, are still in the growth stage and expand, so the timing of the operation is uncertain. The heart cells stop expanding around the age of 15, and there is no difference from that of an adult [10]. Cardiomyocyte transverse diameter has been found to correlate with ventricular wall thickness, ventricular weight, and whole heart weight [11]. Therefore, it seems ideal to perform the operation after the age of 15 years.

## Data Availability

Not applicable

## Abbreviations

CMVL: Cleft mitral valve leaflet
MR: Mitral regurgitation
MICS: Minimally invasive cardiac surgery
